# Shroud waving self-determination: A qualitative analysis of the moral and epistemic dimensions of obstetric violence in the Netherlands

**DOI:** 10.1371/journal.pone.0297968

**Published:** 2024-04-22

**Authors:** Rodante van der Waal, Inge van Nistelrooij

**Affiliations:** Care Ethics Department, University for Humanistic Studies, Utrecht, The Netherlands; Western University Faculty of Science, CANADA

## Abstract

Obstetric violence is an urgent global problem. Recently, several studies have appeared on obstetric violence in the Netherlands, indicating that it is a more widespread phenomenon in Dutch maternity care than commonly thought. At the same time, there has been very little public outrage over these studies. The objective of this qualitative research is to gain insight into the working and normalization of obstetric violence by focusing on the moral and epistemic injustices that both facilitate obstetric violence and make it look acceptable. Following the study design of Responsive Evaluation, interviews, homogenous, and heterogenous focus groups were done in three phases, with thirty-one participants, consisting of ten mothers, eleven midwives, five doulas and five midwives in training. All participants were already critically engaged with the topic, which was a selection criterion to be able to bring the existing depth of knowledge on this topic of people in the field to the fore. Data was analyzed through Thematic Analysis. We elaborate on two groups of results. First, we discuss the forms of obstetric violence most commonly mentioned by the participants, which were vaginal examinations, episiotomies, and pelvic floor support. Second, we demonstrate two major themes that concern practices related to moral and epistemic injustice: 1) ‘Playing the dead baby card’, with the sub-themes ‘shroud waving’, ‘hidden agenda’, and ‘normalizing obstetric violence’; and 2) ‘Troubling consent’, with sub-themes ‘not being asked for consent’, ‘saying “yes”‘, ‘saying “no”‘, and ‘giving up resistance’. While epistemic injustice has been analyzed in relation to obstetric violence, moral injustice has not yet been conceptualized as a fundamental part of both the practice and the justification of obstetric violence. This research hence contributes not only to the better understanding of obstetric violence in the Netherlands, but also to a further theorization of this specific form of gender-based violence.

## 1. Introduction

*Of course, there is a definition of what obstetric violence is, but I don’t think we can just look at obstetric violence in itself, it has to be this intersection of looking at epistemic violence and… yeah. It’s all hanging together, and you can only take little threads, sort it one at a time and kind of critically shine a light and analyze these things*.

*-a mother*

Obstetric violence (in Dutch: *Obstetrisch geweld*) is not a common term in the Netherlands. The Royal Dutch Organisation of Midwives (*KNOV*) and the Dutch Society for Obstetrics and Gynaecology (*NVOG*) do not mention the term, nor synonyms of the term, within any official documents, statements, or guidelines. Before 2020, there were no scientific articles on obstetric violence, or obstetric mistreatment and abuse in the Netherlands. Research on people’s traumatic birth experiences did acknowledge that some forms of communication caused traumatic birth, rather than the unfolding of the event of birth itself. Factors such as a lack of informed consent, lack of communication and unilateral decision making were reported [1–3]. These were not connected explicitly connected, however, to the already existing global critique on obstetric violence, mistreatment, and abuse. Recently, several articles on the occurrence of obstetric violence, mistreatment, and abuse in the Dutch context have come out [4–9]. In these articles, it is shown that 54% of parents experience mistreatment and abuse and almost half of the people who got an episiotomy or medication during labour did not give consent for these interventions [5,6]. Notably, first time mothers and people with a migration background have a higher risk of being treated in a way that is upsetting [5]. The lack of public and professional awareness persists, however, while such mistreatment is a clear violation of human and patient rights.

This article aims to better understand the normalization of obstetric violence through a moral and epistemic analysis, as well as to contribute to delineating the problem of obstetric violence in the Netherlands. Because we specifically focus on moral and epistemic injustice related to obstetric violence, it is important to have some context on the awareness of obstetric violence in the Netherlands, which we first discuss below, followed by a brief note on the clinical organization of Dutch maternity care. Then, the existing research on obstetric violence in the Netherlands is elaborated upon, specifically highlighting work on epistemic injustice. Afterwards, we discuss the methodology of the study before we demonstrate the results, to close with a theorization of moral and epistemic injustice related to obstetric violence. We identify both the withholding of knowledge, the dismissal of mothers’ knowledge, as well as conflicting moral understandings between mothers and medical staff on what is ‘justice’ in reproduction. We understand these different moral understandings of justice to function as a mechanism that has as its consequence the continuous dismissal of mothers as moral and epistemic agents during pregnancy and birth, hence preventing both obstetrics and society in general, to take the violations of bodily autonomy and integrity of mother’s and pregnant people seriously [10,11].

### The Dutch context

In general, public awareness of obstetric violence is very low. There is less attention for the subject in the Netherlands than in neighbouring countries such as Belgium, where obstetric violence has been discussed in the Senate, or such as France, where there has been an official governmental investigation. In Germany, several books have appeared on the subject, and in Spain, a law on obstetric violence almost passed. In the Netherlands, however, there is not much public outrage on the subject, although pregnant people are more and more aware of the need for respectful care and the risk of over-medicalization. The awareness that does exist, has not been raised through traditional platforms such as institutions or the media, but through social media accounts of momfluencers, and workshops by activists, mothers, midwives, and doulas. Most effective was the #Breakthesilence (*#GenoegGezwegen*) campaign by the activist group the Birth Movement (*Geboortebeweging)*, wherein people shared their experiences with obstetric violence [12]. Since a couple of years, this action has been coupled by the action *#TakeResponsibility* (*#HandInEigenBoezem*), wherein health care workers confess their culpability or complicity in obstetric violence [3].

After the first #Breakthesilence campaign in 2016, the Birth Movement created a ‘black book’ with all the stories that were shared, asking for more attention to the bodily integrity of pregnant women and their right to informed consent [3]. The report was submitted to the Ministry of Health in 2017 [3]. In their formal reaction they state that they do not believe it to be a large-scale problem, on the basis of their advisory professionals [3]. But ever since recent numbers came out in 2022 and 2023, indicating that obstetric violence is very much a large-scale problem– 54% of parents experienced disrespect and abuse, almost half of the people did not consent to the episiotomy and medication that they got, and the refusal of interventions by patients was overruled in 20–60% of cases [5,6]–this ministerial statement has not been updated, and neither has there been much public outrage which would compel the ministry to do so. Instead, female columnists of major newspapers described the participants of the studies on obstetric violence as ‘wining’ and as being ungrateful and ‘impolite’ about their child being saved [3,13,14].

The term ‘obstetric violence’ did appear in several media-outlets in the last couple of years, for instance in the Dutch newspaper the General Daily (*Algemeen Dagblad*) and on Dutch public radio channel 1 (*NPO1*) [15,16]. Brainwash, a renowned cultural platform, has an article on obstetric violence on their website and a short informative documentary on the term [17,18]. VICE published an article on the violation of women’s right in Dutch delivery wards mentioning the term [19]. The midwifery platform The Wise Voice (*het vroede geluid*) has a long read on obstetric violence and published an English and Dutch informative video on the term [20]. The pregnancy magazine Baby on the Way (*Baby op komst*) for pregnant people made by midwives has a lemma on obstetric violence on their website [21]. And the magazine for professional birth workers Early (*vakblad Vroeg*) has an article on obstetric violence as well [22]. This fragmented media attention dispersed over a couple of years, and neither caused public scrutiny of maternity care, however, nor a response from the broader feminist movement. It also did provoke any statements, working groups or investigations in professional organizations for midwives or obstetricians [3].

As shown in research globally, the consequences of obstetric violence can be severe, and there is reason to believe that this is also the case for the Netherlands [3]. In the #Breakthesilence campaign, short and long-term consequences were mentioned, such as emotional trauma, difficulty to sleep, and tokophobia [3]. Another study shows that 9% of people who give birth in the Netherlands has a very negative or traumatic birth experience [5]. This percentage is the same as the percentage found ten years earlier [23]. PTSD was found in 1.2% of the respondents [23]. There is evidence linking obstetric violence to the occurrence to PTSD and postpartum depression following labour and birth [3,24]. It is important to point out that there can also be negative consequences of a violent birth experience, such as a lack of trust, not being able to have a good birth-experience anymore, feeling betrayed, and many other long-term ramifications, that are not captured within the above numbers since they did not lead to a traumatic experience or PTSD. We know that at least 36% of birthing people, found something about the way they were treated during childbirth “upsetting”, hence important enough–keeping in mind the amount of normalized obstetric violence–to regret the experience [5].

### A note on clinical context

Dutch maternity care is organized differently than in other countries. The system is divided in primary midwife-led care and secondary obstetrician-led care. In case of a low-risk pregnancy, pregnant people receive midwife-led care in the community by a primary care midwife. Women can choose to give birth either at home, in a birth centre, or in a hospital with the primary care midwife as the responsible care provider. Primary care midwives are therefore a strong independent professional group in the Netherlands. In case of risk factors or complications during pregnancy or labour, people are referred to obstetrician-led care, where they are tended to by nurses, hospital-based midwives, general doctors, gynaecologists, and gynaecologists in training. This makes the Netherlands a unique setting to study obstetric violence through a range of maternity care practices. When it comes to experiences of obstetric violence, midwives and obstetricians were mentioned by parents in roughly equal numbers in the #Breakthesilence campaign, indicating that pregnant people experience disrespect and abuse throughout the whole Dutch Maternity care system [3,25]. Several studies on experienced client care provider interaction during labour and birth in the Netherlands did show that people who give birth with a community midwife at home experience more respect, communication, autonomy, and confidentiality in the interaction compared to women who give birth at the hospital with a (resident) obstetrician or hospital-based midwife [3,4,26].

Despite the solid existence of autonomous midwifery care, maternity care in the Netherlands is in many places defined by a top-down hierarchical system in which gynaecologists ultimately decide when someone should be referred to the hospital through local or national guidelines. The decisions of doctors can, in line with obstetrics globally, be described as risk aversive [3,27,28]. If a midwife does not refer someone to the hospital when the hospital thinks they should, this can cause differences of opinion on who is the deciding professional in terms of risk selection. For pregnant people referral policy can be untransparent and confusing–one moment they are in midwifery care and the next moment they have been taken over by obstetric care–and they have limited control over these decisions, especially when they do not have much social privilege [3,29–31]. Consequently, pregnant people wishing to take more risks than is recommended in the official protocols, are often seen as a problem. In an attempt to battle this, prominent birth activists, such as the independent midwife Rebekka Visser and the gynaecologist Gunilla Kleiverda, have made a professional guideline for care outside of guidelines, which is for pregnant people, and care workers willing to go outside of the guidelines, an important tool to advocate for their rights and make such care possible [3].

### Scientific studies on obstetric violence in the Netherlands

Two quantitative studies on obstetric violence were conducted in the Netherlands based on one survey in which approximately 13.000 people participated [5,6]. 54.4% of respondents reported at least one form of disrespect and abuse. ‘Lack of choices’ (39.8%) was reported most, followed by ‘lack of communication’ (29.9%), ‘lack of support’ (21.3%) and ‘harsh or rough treatment/physical violence’ (21.1%) [5]. 42% of the people who got an episiotomy did not give consent, 47% of people who got medication did not give consent, and around 36% did not give consent for electronic foetal monitoring or a foetal scalp electrode. Of the people who refused an intervention, many were overruled, respectively 40% in the case of vaginal examination, 60% in the case of medication, and 40% in the case of an episiotomy [6].

In 2020, a qualitative content analysis was performed to investigate the stories shared in the #Breakthesilence campaign, based on the typology of Bohren [25,32]. Situations of ineffective communication, loss of autonomy and lack of informed consent and confidentiality were mentioned most often. Five main themes were established: ‘Lack of informed consent’, ‘not being taken seriously and not being listened to’, ‘lack of compassion’; ‘the use of force’, and ‘short and long term consequences’. These themes were often described in combination with a lack or losing control, fear, being objectified and being humiliated. ‘Left powerless’ was the overarching theme; people felt that power was taken away from them, or they experienced difficulties maintaining control due to situations that occurred. In around one fifth of stories, a form of use of force was described, mostly during the active stage of labour and in relation to interventions being carried out [3,25].

In 2020–2021, a cross-cultural thematic analysis was done on the effect of obstetric violence and obstetric racism in the training of midwives and doctors [7]. Students’ curricular encounters in two colonially related geopolitical spaces, South Africa, and the Netherlands, were amplified to highlight global systemic tendencies that push students to cross ethical, social, and political boundaries towards the mother they are trained to care for [3]. Obstetric violence was identified as part of students’ rite of passage in becoming professionals. The following violent instances within the rite of passage that led to the reproduction of obstetric violence were differentiated: ‘Having to adapt the goals, norms, and values of the obstetric institution that instrumentalize the mother’; ‘establishing subjectivity through assertiveness, and competition’, ‘learning at the cost of mothers’, ‘colluding in explicit obstetric violence, obstetric racism, and sexual violence’, ‘traumatic experiences’, ‘complicity’, ‘balancing guilt with numbness’, and ‘taking responsibility at the cost of mothers’ [3,7].

Epistemic injustice is broadly defined as an unequal relationship in the domain of knowledge, meaning either that some have less access to knowledge than others, that some people are regarded as epistemic agents and others not, or that authoritative knowledge is used to manipulate someone. The analysis of the #Breakthesilence campaign showed that in almost half the stories people report being ignored and/ or not taken seriously, for instance by care workers not talking *to* but *about* them while they themselves are present in the room [3,25]. In 2023, an auto-ethnographic study was published wherein the epistemic component of obstetric violence was specifically investigated [9]. This was the first study on obstetric violence in the Netherlands that centred epistemic injustice as an important part of obstetric violence. Through a narrative analysis of four auto-ethnographic experiences with birth and miscarriages in the Netherlands, four forms of epistemic injustice in Dutch reproductive care were laid bare and analysed through the theoretical literature on the subject: ’Hermeneutic injustice’; ‘testimonial injustice’; ‘gaslighting’; and ‘wilful hermeneutic ignorance’ [3,9]. Hermeneutic injustice is when a pregnant person does not have the right knowledge or discourse to understand and explain the obstetric violence being done to them. Testimonial injustice is when a pregnant person is not believed or not taken seriously with regards to the violence done to them. Gaslighting is when the knowledge of the pregnant person is doubted in such a way that it is insinuated that the pregnant person is crazy or a bad mother [9,33]. And, in the words of Gail Polhaus, ‘wilful hermeneutical ignorance occurs when dominantly situated knowers refuse to acknowledge epistemic tools developed from the experienced world of those situated marginally. Such refusals allow dominantly situated knowers to misunderstand, misinterpret, and/or ignore whole parts of the world’ [34].

## 2. Methods

### Participants and sampling

The study design is Responsive Evaluation (RE) (Abma & Widdershoven, 2005), adapted to care ethics (Visse, Abma & Widdershoven, 2015). Additionally, this study was specifically designed according to the insights of Standpoint Theory which regards experiences of marginalized people a source of knowledge (Harding, 2004). The epistemic value of the knowledge of marginalized groups can remain unrecognized, which is why it is important in the study design to pay attention to how this is brought best to the fore (Harding, 2004). The study design was also adapted according to the insights of care ethics, which holds that theory and empirical data are always constituted dialectically and cannot be objectively separated (Leget et al., 2017).

Thirty-one participants were recruited by the first author. The participants consist of ten mothers, eleven midwives, five doulas and five midwives in training. Most of the birth workers are also mothers. People were contacted through personal networks, as well as through the activist organization the Birth Movement (*Geboortebeweging*). Participants were also recruited via snowball recruitment. Sample criteria were extensive knowledge (either scholarly, experiential, embodied, etc.) of obstetric violence, mistreatment in maternity care, violation of rights during pregnancy and birth, and/or active engagement with activism or alternative forms of care. This means that all participants were critical (ranging from quite to very critical) of Dutch maternity care, and that they had experience with, and ideas on, how care for birth can be better and more emotionally safe. To be able to include a breadth of perspectives, analyses, and practices, attention was paid to establishing a diverse group of participants in terms of both identity, such as people with and without a migration background, and practices, such as community midwives and caseload midwives. Some participants engaged in direct activism, for instance as part of the Birth Movement, others in reading and study, some for themselves and others as scientists and educators, while others make art related to the subject, and again others offer alternative forms of pregnancy and birth care, such as care outside professional guidelines.

It is important to note that this study, being a qualitative one, does not aim to represent the general experiences of the Dutch population with regards to Dutch maternity care. For this purpose, we refer to the work of Marit van der Pijl discussed above, who did a mixed methods analysis of a large group of participants based on a questionnaire and a study of the #Breakthesilence campaign [4–6,25]. In the present study, we have done in-depth interviews and focus groups with critical and engaged people from the field, to be able to get a better understanding of the already existing practical knowledge on the main patterns enabling obstetric violence, and the potential solutions. Participants were selected on their ability to think critically about obstetric violence. Therefore, all participants already thought that obstetric violence is a serious problem before participating in the study. Our participants thus do not present the average Dutch maternity care population, since there are of course many midwives and mothers in the Netherlands who have never heard of obstetric violence, or who do not think that it exists or is a serious problem. We have deliberately selected our participants on their ability to analyse the root causes of obstetric violence, in order to tap into their critical capacity to arrive at insights with regards to the current problematic situation in obstetric care wherein 54% of people experience disrespect and abuse, and that many mothers, midwives, midwives in training, and activists describe as obstetric violence [5].

### Data collection

Following the method of Responsive Evaluation, data were collected in three rounds: individual interviews, homogenous focus groups, and heterogenous focus groups [35]. Interviews were conducted by the first author in 2020. Recruitment started on the 15^th^ of June of 2020 and ended the 1^st^ of December of 2021. Because of the Covid 19 pandemic, almost all of them were online. Some participants did not want to participate online, these interviews were held in person. All interviews lasted a median of a bit over two hours and were in-depth. They were semi-structured based on familiarization by the interviewer with the interviewee’s thought as expressed in previous conversations, publications, Facebook discussions, etc. There was a minor interview guide with themes and questions based on the familiarization of the reviewer with the thoughts and views of the participants; the guide was only applied when the need arose to lift the conversation to an analytical level, there were no additional external themes added to the topic list. Open, non-directive formulations that were consistent with the interviewee’s own vocabulary were used. Notes were made during the interviews which were as well recorded, anonymized, and transcribed ad verbatim. Two recordings were lost due to a technical failure, but the notes were still used. After the first round of individual interviews, they were preliminary analysed by the first author, and a thematic analysis was sent to the participants for a member check [36]. The participants were given the opportunity to give feedback in writing or during the following focus groups.

Homogenous focus groups were conducted at the end 2020 and beginning of 2021 [35]. For organizational reasons (primarily finding a suitable timeslot for the entire group), multiple homogenous groups were formed, six in total (two groups of four midwives each; one group of five doulas; one group of four midwives in training; one group of three, and one of four mothers). The focus groups interviews were semi-structed with a topic list based on the analysis of the individual interviews. They lasted a median of almost three hours. They were all done online, recorded, anonymized, and transcribed ad verbatim. The homogenous focus groups were then again preliminary analysed, and this thematic analysis was sent to the participants for a member check [36].

Heterogenous focus groups were conducted in 2021 [35]. Three heterogenous focus groups were done since it was difficult to get more people together on the same date (two focus groups with five participants, and one with six). The groups were all mixed. The focus group discussions were semi-structured with a topic list based on the analysis of the homogenous focus groups. They were online, recorded, anonymized, and transcribed ad verbatim.

### Data analysis

The analysis was conducted by the main author through Atlas.ti under supervision of the second author. The Thematic Analysis (TA) method was used [36]. TA has 5 phases before drawing up the results: 1) familiarizing yourself with the data; 2) generating initial codes; 3) searching for themes; 4) reviewing themes; 5) defining and naming themes [36]. The first and second phase were done after every step part of the RE study design. After the interviews, the homogenous focus groups, and the heterogenous focus groups, we familiarized ourselves with the data and sent an initial interpretation with some initial codes and themes back to the participants for feedback. After all the data were collected, we again familiarized ourselves with the dataset as a whole by reading through the transcripts again and comparing the different data that came out of the different phases of the RE design. We then searched for themes, and found many, due to the amount of data we collected. For this study, we therefore decided to focus on the moral and epistemic dimensions of epistemic violence, and not on other causes and mechanisms of obstetric violence (for instance more historical and cultural theories on why obstetric violence exists), nor on activist resistance against obstetric violence, on obstetric racism. These causes and dimensions of violence in obstetrics are all discussed in separate studies. After making this decision, we reviewed all the themes and went back to the data, to see if this differentiation made sense. After confirming the above, we came to the last step of defining and naming themes for this specific study.

### Positionality

During the study, we have been aware of our positionality. Rodante (she/they) is a cis gender white middle-class practicing midwife in Amsterdam and a PhD-candidate in care ethics. Inge (she her) is a cis gender white middle-class married mother of three who works as an associate and endowed professor in two universities respectively. We recognize that our positionality in this study influences the way we understand the data. Therefore, we do not claim to be objective. Rather, we believe that our proximity as researchers to the standpoint of the participants, as both a midwife and a mother, makes it possible for us to deeply understand and represent the data through our subjective identity and positionality, in line with standpoint theory, on which our study design is based.

### Ethical considerations

This research was evaluated and approved by the ETC of the University for Humanistic Studies in January 2021. The METC of the University of Utrecht decided in 2019 that the Dutch Medical Research Involving Human Subject Act (WMO) did not apply, as participants were not patients but mentally competent citizens, and participants were not subjected to treatment or required to follow a certain behavioural strategy as referred to in the WMO (art.1b).

All participants were given an information sheet prior to the study and room for questions at the beginning of the individual interviews. Privacy details were discussed and their right to withdraw at any moment was made explicit. They all gave written informed consent to their participation in the study and most also for the anonymized publication of the interviews and focus groups in the DANS archives.

## 3. Results

Below, we present two sets of results from this study. First, we list the most common forms of physical obstetric violence in the Netherlands that come out of this study, respectively ‘vaginal examination’, ‘episiotomy’, and ‘pelvic floor support’. These are not the only forms, but these are the ones most mentioned by the participants. They should thus be understood as forms of physical mistreatment that come to the minds of the participants most often when discussing obstetric violence. We share these results so that the reader can get an idea of what the participants understand mostly under physical forms of obstetric violence. These forms of obstetric violence also come back frequently in the second set of results. The second set of results concerns the main themes of the analysis of obstetric violence as related to moral and epistemic injustice. The main themes can be read as practices that facilitate, reproduce, and justify, the forms of physical obstetric violence in the first set of results. The two main themes established when it comes to epistemic injustice are: ‘Playing the dead baby card’, with the subthemes ‘as shroud waving’, ‘as hidden agenda’, and ‘as the normalization of obstetric violence’; and ‘troubling consent’, with the subthemes ‘not being asked for consent’, ‘saying “yes”‘, ‘saying “no”‘, and ‘giving up resistance’.

### 3.1 Most common forms of physical violence

In this study, most cases of physical obstetric violence consist of interventions done without consent, without sufficient consent, or under explicit refusal. Neither in this study, nor in the ones from Van der Pijl, physical violence was mentioned outside of medical interventions, which we do come across in other countries, such as random pinching or slapping [37,38]. The examples below are the physical forms of obstetric violence that came up most often in conversation (at least in three individual interviews and one focus group), listed from most to least often, and were recognized by the participants as interventions that happen on a regular basis. Other forms of obstetric violence were recounted as well, like getting an IV or medication without consent, being stitched without proper anaesthesia or having to wait very long for anaesthesia, or inspection of the anus after labour without sufficient consent and information, but less often. Interestingly, all forms below have to do with unconsented, unwarranted, or unwanted vaginal penetration.

The first physical form of obstetric violence, vaginal examinations, was mentioned in nine individual interviews and two focus groups. The second one, episiotomy, was mentioned in eight individual interviews and two focus groups. The third and last one, pelvic floor support, was mentioned in three individual interviews and one focus group. Notably, the participants were not explicitly asked to list forms of obstetric violence that they had experienced or encountered, since the interviews were focused on the causes of, and solutions to, obstetric violence. These are hence all experiences that the participants brought forward as exemplary cases of obstetric violence on their own account. For each form of obstetric violence, one citation is provided.

### Vaginal examination

*It’s always annoying when people touch you. And if someone touches you down there, you just feel like an object, you know? […] At one point, they told me: "we have to let the next shift know how far you are". And then I said "do you really have to?" […] Until then I said every time: "I do not need to be examined, we will just see". And then they said again: "we need to know how far you are for the next shift" […] I told her beforehand that "if I say stop, you have to stop". And then I said stop, but she just wanted to know how far I was. So, as I said stop, I felt her fingers go further and I felt her fingers spreading. Later I said to her "I said no, but you continued." When she left, I had to cry. (Mother 10)*

Unconsented, unwarranted, or unwanted vaginal examinations is the most common form of physical obstetric violence listed in this study. As a procedure in a highly intimate region of the body part which might have been part of experiences with sexual violence, it is experienced as very invasive. Mothers complain about multiple people doing vaginal examinations during their labour. The participants, both mothers and midwives, say mothers often do not know that they can refuse vaginal examinations, neither do they know that their regular performance is a highly contested and often not evidence-based intervention [39]. While only 7% of participants in Van der Pijl’s research lists that were *not* asked for consent for vaginal examinations, 60% of those who refused the examination said that their refusal was overruled [6].

### Episiotomy

*A few moments later, the doctor pulled out the scissors, and the woman cried out: “No, I don’t want to be cut.” And then the doctor went to her and said: “You and I both know that the only thing that is important, is that the baby gets out alive.” And then she said: “But I don’t want to be cut, I want to do it myself.” And even the midwife that was there said afterwards: “There was really no need to cut, also not from the monitor, the baby was doing fine, there was no need to cut.” […] And the doctor said to her: “Okay, I will give you two contractions. And if by then, the baby is not born, I will cut.” […] And then, after one contraction, instead of keeping her promise, she made the cut. (Doula 4)*

Episiotomies, when done unconsented, are probably the most well-known form of obstetric violence. It is a cut through the vaginal wall and the muscles of the pelvic floor. As in the quote above, the ‘dead baby card’, a form of shroud waving to get the mother to consent to proposed policy, is often played in situations of obstetric violence, both to convince the mother, as well as to justify the obstetric violence which is inflicted. The play of the dead baby card will be further elaborated upon in the second set of results below. In the Netherlands, the only right indication for an episiotomy is when the life of the foetus is in danger. But there is an enormous difference in the number of episiotomies between hospitals and between birth workers in the Netherlands, ranging from 14% till 67% depending on whether the birth was midwife- or obstetrician-led and depending on which hospital one is in, and an 8% till 48% difference depending on the individual care-worker within the hospital [40,41]. This variation indicates that episiotomies are done way more frequently than only in cases of life-threatening emergencies, and that the chance of getting or not getting an episiotomy depends more on external circumstances than on the process of birth. Other research furthermore found that 42% of participants who had an episiotomy, were not asked for consent, and that 25% of those who refused the cut, were overruled [6].

### Pelvic floor support

*Pelvic floor support is something that I find very intense… that you just keep putting your fingers in someone’s vagina every contraction, just like that, and hard too…. […] Especially if you also put someone on their back in a bed. I always have to think of an insect, when I see it, you know? An insect that lies on her back, on her shield, incapable to move. (Midwife 11)*

Pelvic floor support is an unknown form of obstetric violence, almost never listed in any of the international literature. It was also not part of the other Dutch studies on the subject. Pelvic floor support is a technique where the birth worker during the second stage of labour inserts two fingers of each hand into the vagina to “pull” the pelvic floor away during each contraction, to create more space for the head, often performed without (sufficient) consent. It is an intervention done to speed up the pushing phase and sometimes to make clear to the labouring person in which direction to push. It is an invasive intervention, given the weight the birth worker throws behind stretching the pelvic floor with their hands. Sometimes, when done with not enough lube or the wrong gloves, or when simply done too strong, it can cause ruptures of the vaginal wall.

### 3.2 Moral and epistemic injustice

Many different forms of moral and epistemic injustice are mentioned in the study. We choose to zoom in on two specific practices in which both moral and epistemic injustice come to the fore, namely ‘playing the dead baby card’, with the sub-themes ‘as shroud waving’, ‘as hidden agenda’, and ‘as normalizing obstetric violence’, and ‘as troubling consent’, with sub-themes ‘not being asked for consent’, ‘saying “yes”, ‘saying “no”‘, and ‘giving up resistance’.

#### 3.2.1 Playing the dead baby card

Something that was mentioned often by the participants in discussing obstetric violence was the moral priority of the baby over the mother, specifically coming to the fore in practice through what is known as ‘playing the dead baby card’, a form of shroud waving specific to obstetrics. This practice happens globally in maternity care, consisting of care workers either not explaining well the precise risk of the baby dying (for instance, when it is said that the risk that the baby dies ‘doubles’ when it goes from 0.01% to 0.02%), or simply exaggerating the chance of the possible death of the foetus to get the parent to comply with their proposed policy. But the dead baby card can also be played as an implicit form of shroud waving, appearing as an indirect accusation or a hidden agenda, or it can be used after labour to normalize or justify the obstetric violence that took place during birth. In all three cases discussed below, an implicit moral understanding of birth workers of what is justice, or the right thing to do in matters of reproduction comes to the fore, which is in conflict with mothers’ own configuration of reproductive–e justice.

### As shroud waving

*She was the head of the department*. *[…] You saw that she really feels her place of power*. *[During our prenatal consult]*, *she started to cry*. *She teared up and then she said*: *‘I am sorry*, *I know it is not professional*, *but I have just seen a lot of dead babies*.*’ Yeah… I am not shitting you*.*‬ And I thought*: *she can’t help me*. *This is obviously not a place where they have their shit together because the top of the gynaecology department is crying in front of me*. *That’s not what I had in mind*, *you know*. *But my partner*, *he bought it*. *[…] And he said*: *“I don’t want the baby to die*. *I don’t want the baby to die*.*” And I felt so manipulated*. *I felt so coerced*. *(Mother 1)*‬‬‬

In the quotation above, the dead baby card is played directly and theatrically. It was used as the last resort by the doctor to get the mother to comply with hospital policy. This particular discussion was about the fact that the mother did not want to be induced when recommended by the guidelines. Note that induction rates in the Netherlands are highly dependent on the region in the Netherlands, ranging from 14.3% to 41.1% [42]. The mother was additionally considered high risk due to a high BMI, so they wanted her to birth in the hospital while she wanted to give birth at home. And while there was a medical reason to be induced because of a slight increase in risk, it remained, of course, the mother’s decision. Due to the mother’s desire to give birth at home despite a slight increase in risk, the midwife wanted her to speak with a gynaecologist, which is something that is recommended often by primary care independent midwives when people have alternative care plans. And while the mother did not want to herself, in the end she made an appointment with the gynaecologist to satisfy the midwife. At the time of their meeting, the mother had already made her decision and already had to defend it multiple times to her own midwife. She experienced the encounter with the gynaecologist as manipulative. Five days later, she gave up her resistance, since she was tired, scared, and without support, and she went into the hospital. There, they started the medication for the induction without her consent: “at one point I went to sleep, and while I was sleeping they started contractions.‬”‬

Multiple forms of shroud waving were used to get the participants in this study to comply with proposed interventions. Doulas and midwives in training often reported shroud waving as a form of obstetric violence. Midwives, however, also recognized the dilemma between trying to convince someone of proposed policy based on risk determination, and the thin border towards manipulation.

### As a “hidden” agenda

*The first thing [the psychiatrist of the hospital] said [during the prenatal consult] was: “So, you had a psychosis three times?” He looked at us very intensely. “That is extremely bad, of course you do not want that to ever happen to you again.”[…] The thought behind it was that in madness I could kill my child, and that must be prevented. I think this is the thing that is not spoken about but what [birth workers] find the most scary. That they would have to deal with a mother who would kill her own child and that they could have prevented it. […] So, they will ensure that a path is followed in which this is prevented by any means necessary. […] They put you under supervision in an institution, including the flattening of my mind [by medication during pregnancy, birth and postpartum] and everything. (Mother 2)*

This mother had experienced psychosis in the past, which made her high-risk for another psychosis. She was off medication for a long time, however, and she and her husband had a lot of experience with managing her mental health. She had a deep desire to experience childbirth consciously, without medication that, based on her own experiences with it, “flattens” her mind. She had her own familiar psychiatrist who also thought it would be possible to give birth without medication. The consult with the psychiatrist from the hospital was planned, the parents thought, to discuss how she could go through pregnancy and childbirth without using medication. The hospital psychiatrist, however, refused to think along with them. Instead, the possible death of the baby–or, more precise: the possible murder of the baby by the mother–was given as an implicit reason for her to start medication. The fact that the dead baby card was not played openly, but that the psychiatrist danced around it as a kind of taboo, can be interpreted as making the situation even more stigmatizing. Not only does the implicit reference to the baby’s life function as a way to manipulate the mother into taking medication she does not want, it also dismisses her as an epistemic agent during the conversation itself, as if the hypothetical death of the baby during her hypothetical psychosis already comprised her epistemic capabilities at the moment of the consultation. Just as in the case above, this happened while the parents had no need for more information on the benefits of the hospital policy, and they had already been going to multiple consults having to defend themselves.

By now, this mother has had two children without taking any medication, and without having had a psychosis surrounding their births. The parents have carefully managed the pregnancy, childbirth, and postpartum period together with community midwives, maternity care nurses (kraamzorg), social workers, and her own psychiatrist.

### As the normalization of obstetric violence

*There’s a whole normalization throughout society about this being okay and normal for you to be treated this way. In my case, there were three people [birth workers] present at the birth of my kid. All three apparently thought it was super normal what happened while I was being traumatized. […] And then [at the consultation] afterwards, [the gynaecologist] was being very nice while turning my story around completely, really gaslighting. […] He said: “Of course you experienced that as horrible, but yes, it was necessary, because otherwise we do not know what would have happened…” (Mother 9)*

After experiencing multiple instances of obstetric violence, during a severely traumatizing birth, this mother goes to the hospital a couple of weeks later to talk about her birth experience. She had many questions about everything that had happened. But rather than taking responsibility for the traumatizing care that was given, the doctor continuously suggested that it was absolutely necessary for the procedures to have gone this way, otherwise the baby might have died. In the quote above, he again implicitly plays of the dead baby card during the evaluation talk of the birth, in order to both normalize and justify the obstetric violence during the birth. The doctor did not give evidence-based reasons why they handled the birth this way, and hence did not answer the mother’s questions, but simply insinuated that otherwise her baby would be dead. This normalization of obstetric violence through the play of the dead baby card, she calls “gaslighting”: her experience is acknowledged but in the same sentence not only made irrelevant, but pathologized, the implication of his comment being that only bad or mad mothers would have wanted to risk the life of their child, hence making her question her own judgement and her motherhood by merely wanting to discuss what had happened during her labour, which had been so traumatic to her.

#### 3.2.2 Troubling consent

While research in the Netherlands shows that almost half of the mothers who received medication or an episiotomy did not give consent [6], in this study it additionally comes to the fore that even when mothers do give consent, it is often the case that this consent was illegitimately obtained; achieved through a form of moral or epistemic injustice. All groups of participants recognized this ‘trouble’ with consent, especially the midwives. The troubling of consent is described through the sub-themes ‘not being asked for consent’, ‘saying “yes”‘, ‘powerplay’, ‘saying “no”‘, and ‘giving up resistance’.

### Not being asked for consent

*Well, I believe the gynaecologist said very simply: “what you have in your birth plan, you cannot do here. If your delivery is medical, we want to check the heart, so you need to have an ECG.” I said: “yes, but what if I just do not want to?” “No, you just have to.” I said: “but I really don’t want to.” She said: “Well, if it comes to that, you will just have to.” I looked at my husband like: who is going to do something about this? Something is about to happen that I don’t want. I said: “but I do not give permission for this.” She said: “Well if you come here, you have no choice. And the chances are very high that you end up here, because with a first child 70% of women end up in the hospital, so I would prepare myself for it, if I were you.” […] I still don’t understand how someone can just say: we’re going to do something with your body that you do not give consent for. (Doula 3)*

To this mother, the doctor blatantly said that it did not matter whether consent would be given or not. It was going to happen the way the doctors wanted to anyways. The mother was shocked, and she objects that something is going to happen against her will, but this has no effect as it does not seem to matter. Not only her consent, also her objections, based on her own moral and epistemic views, were silenced. Many participants reported that pregnant people were not asked for consent. This is congruent with the approximate 36% who got electronic foetal monitoring or foetal scalp electrodes, and the 42% of parents who got an episiotomy, and the 47% who got medication, without being asked for consent [6]. While not asking for consent can sometimes be understood as a form of presumed or opt-out consent (which are considered to be insufficient forms of consent), there were many stories like the one above where it becomes painfully clear that there seems to be no need for consent from the mother at all.

But there are also cases in which not asking for consent is made less explicitly made clear, for instance in cases of opt-out or presumed consent. Rather than acknowledging that the responsibility to obtain consent lies with the medical professionals, in cases of presumed and opt-out consent, the responsibility for consent is given fully to the mothers, presupposing that all mothers are vocal enough to advocate for themselves:

*It’s the same in the whole debate around rape and what rape is. Often, we say to each other "she didn’t say ’no’." (Midwife 7)*

In making the comparison with rape, the midwife above explains that the violation in not asking for consent is severe, and that it is a bad excuse that “she did n’t say ‘no’”. She flags that there should be a moral awareness that many of the interventions done during birth are simply not interventions where opt-out or presumed consent suffices, due to their intimate nature, just like with sexual intercourse.

There being no need to ask for consent is, as flagged by many participants, racially stratified. It depends on who care workers have in front of them whether they deem asking for consent necessary. Interestingly, it is not the high educated middle-class people that are to be expected to be able to advocate for themselves more easily in cases of presumed or opt-out consent, who are asked less often for consent. Midwives, doulas, and especially students signal that exactly the people for whom it is harder to advocate for themselves, are asked for consent less, and that they are in that sense taken advantage of:

*I have a feeling that maybe migrants think more often: oh, it’s normal for two people to do an internal examination, or, you know, that must be normal, or that’s how it should be, you know.[…] People who, for example, are highly educated and come from a certain environment and perhaps have a medical education themselves, they are more on top of it, like: “Wait a minute, you know, I understand very well what is happening here.” And migrants, or maybe people who are less well educated, or not medically trained, yes, they are less assertive about this. So, they also let a little more happen. While I think: we should not lie to anyone. (Midwife in training 3)*

The epistemic inequality that is established by keeping parents in the dark, or that exists due to social and economic inequalities, language barriers, or difference in culture, is hence exploited and reproduced. Participants explained as well how other stigmatized identity characteristics or experiences besides having a uterus are used to justify making even less of an effort to establish a relation of knowledge exchange wherein the proper obtainment of consent can take place:

*I told them: “I have a history of sexual violence, so I feel very vulnerable and very afraid of what’s going to happen.” And looking back I really feel like disclosing this did not help me at all, instead it turned against me. The fact that I had said that made that they took me even less seriously, and apparently made it even more difficult for them to talk to me, to relate to me or to have any idea what to do. It was awful, it was awful. (Mother 6)*

This quote, but also the quote above where the student midwife discussed the exploitation of epistemic inequality, is one of the many examples where participants were taken even less seriously on the basis of their identity.

Not asking for consent comes forth from the conviction that consent from mothers is not always needed, as made explicit in the first quote that was discussed, influenced by the level wherein the mother is taken seriously as an epistemic actor based on identity characteristics. This makes it increasingly problematic to see *not* asking for consent as a form of presumed or opt-out consent.

### Saying “yes”

*It’s so incredibly easy to say to someone, I want to do a vaginal examination. And then they almost always say yes. (Midwife 10)*

Even if consent is asked, it can be more of a formality than a real question, especially since birth workers are used to people responding affirmatively. Many mothers in this study recount that they gave consent because they did not know that it was an option to say “no”. Mothers also explain that they sometimes say yes since they felt that it was not really an option to say no:

*you have to give permission for everything yourself. But "yes" is the only correct answer. (Mother 5)*

This quote is congruent with the recent numbers that show that refusal is overruled at a rate of 50% or more, in the case of vaginal examination, augmentation of labour, and electronic foetal monitoring [6]. Only the caesarean section, with a 13% overruled refusal rate, scores below 20% of all the interventions during labour in which refusal was overruled [6]. Considering that a caesarean section against one’s will when one explicitly says no is an extremely invasive form of obstetric violence, this is still a shocking number.

The epistemic authority of the medical professionals, can thus result in false obtainment of consent, even when consent is formally asked and mothers have explicitly said “yes”, either due to the lack of knowledge of mothers that it is possible to refuse proposed policy, or due to their interpretation of the situation as one wherein it is not really possible to say no. Just as in the case of not being asked for consent which is often framed as presumed or opt-out consent, also consent obtained through an explicit “yes” without sufficient counselling, should not be confused with true informed, opt-in consent, as it often still based on a negation of the mother as an epistemic agent–a form of epistemic exploitation that midwives are very aware of. There is an additionally certain moral ignorance at play here, where care workers are either not aware of the moral need for consent in these kinds of intimate situations, or where it seems to be ok to not take the need for true informed consent too seriously [43].

### Saying “no”

*I yelled very loudly “NO!” [during a prenatal consult at the end of pregnancy]. I got off the bed. I tried to run to the exit. They pushed me back onto the bed and took off my clothes. […] I felt my baby kick. I thought everything was going well. I can still see myself crawling off that bed, but I just got pushed back. And that was it. End of story. And then I sort of went into shock […] No one has ever stabbed me to do what they thought was right without letting me say something about it. And yes, it cannot get any worse than this. They cut me open, opened me up. And what I loved most, they took out of me. And the bizarre thing is, I know that all those doctors would say that it was a very successful operation. While all I think is: what have you done? My human rights were violated, but I am the only one who thinks so. (Mother 4)*

In the quote above, the mother recounts her unconsented emergency caesarean section to which she explicitly objected. As mentioned above, in the Netherlands 12% of the refusals of caesarean sections during labour are overruled [6]. The doctors believed the caesarean section to be necessary based on the electronic foetal monitoring. The mother knew that there was nothing wrong with her baby, since she felt the baby kick, and she tried to tell that to the doctors. The doctors thought that they were doing the right thing by saving the baby’s life in an emergency. Eventually, the baby was born healthy and not in a critical condition, so in this case the mother was right. The negation of her objection, led to direct physical violence. Consequently, she went into “shock”, and had a highly traumatic experience. Here is becomes clear how moral and epistemic injustice work together: it was the negation of the mother’s knowledge that made the caesarean section seem highly necessary, and it was the moral believe that saving the life of the baby, even when the mother objects to the operation, is the right thing to do. Due to the combination of these two factors, the doctors were able to think that it had been a successful operation even though the mother turned out to be right about the condition of the baby and was severely traumatized. Although the complete negation of an explicit objection is rarer than not being asked for consent, most participants did recount a story wherein an explicit objection was overruled.

What is important to note, is that saying no can also occur after consent was given or presumed. Consent can be taken back, either with an explicit no, or through another utterance which indicated the retrieval of consent, but this second ‘no’ is often overruled. The student below explains that she regularly comes across situations wherein consent was asked for pelvic flour support in a euphemistic way, namely as “helping” the woman a bit to push. And that then, when the mother clearly indicated to be in pain, consent was not obtained again, although the student experienced these utterance as clear ways of saying “no”:

*When a woman says ’ouch’ or something like that, then they act very empathetic, they say: “yes, I know it hurts, but I have to help you.” But they do not stop or ask if they can continue. (Midwife in training 3)*

Many of the midwives also described the experience that consent is given, but afterwards they notice in the body language of the mothers that she is not consenting anymore:

*If you ask someone "can I cut?" and she says yes, but she pushes her legs together; I do not call that consent. […] You can say “yes, I want to have sex with you now” and say five minutes later: “I do not want to anymore”. Does it no longer count because you already said yes? The same applies here, I think it is the same kind of discussion. […] (Midwife 7)*

While some midwives in this study used to continue in situations like this, most said that they are currently trying to listen extremely carefully to body-language and everything that is being said. Especially listening to body language, and utterances like ‘ouch’, is important because many mothers do not dare to explicitly take back consent. Midwives attempt to be aware of signs of taking back consent or “false” consent. One midwife describes this as: “if you do not want to cross someone’s borders, you have to be aware in each fibre of your body what her borders are.” (Midwife 11)

### Giving up resistance

*My resistance just ran out at some point, I think. So that’s why I agreed to it. […] But I was angry, I was sad. Actually, it was not ok. [..] It did not feel right to me. I actually could not support the decision to go into this plan. […] so that we went down that road, it was not with conviction on my part. I mean, it was how it was, my resistance ran out. And then the gynaecologist had such a complicated story on why EFM was so important. […] I did not really understand it either. And then I thought: well, it must be my fault that I do not understand it then. (Midwife 6)*

Here, a midwife tells the story of her own birth. In the last weeks of pregnancy, she had lost the argument with the gynaecologists. This midwife ran out of resistance within an exchange of knowledge and arguments, feeling at some point like she “lost”, and she gave in. Her resistance hence ran out due to a form of epistemic injustice: she recounts not being able to follow it anymore, while she, in contradiction to most mothers, has extensive knowledge on the subject being a senior midwife. So even when one has a lot of experience with childbirth, has a midwifery diploma and is already practicing as a midwife for many years, an epistemic hierarchy can be inserted into a relation of more or less equal epistemic agents, in order to push the mother to consent to the policy proposed.

Another midwife recognizes this push to give up resistance as a kind of “powerplay”. She describes:

*Consent is really just a negotiation of someone’s boundary. […] A woman suffers from the fact that she lacks certain knowledge and experience needed to assess the situation, and she does not know whether I am sincere. When I want to do something, I can exaggerate that. […] I think that as a healthcare provider you ultimately have the power and can therefore use that as violence, and as a woman you have very little to defend yourself against it. It is dangerous, because I can say anything, I can say anything I want, she has to trust that what I say is correct. (Midwife 7)*

As we also saw above, many midwives voiced concerns about the sincerity with which they obtain consent. They explained that the trouble with consent is based on a power difference due to a difference in knowledge, or justified by a difference in knowledge. In the quote above, it becomes painfully clear how this difference in knowledge can be used to obtain consent on false grounds, and how that directly leads to physical interventions, hence troubling the right to true bodily self-determination.

In the quote below, the troubling of consent and the play of the dead baby card come together, and ultimately make the mother give up her resistance:

*I have seen that birth workers just keep insisting. That someone says: “no, I don’t want a needle just in case.” And they say: “Yes, but if it does end up being a caesarean section, then it can really be very… it will take us way too much time. That can really make a difference from life to death.” And the mother says “Yeah, but I really just do not want it.” And they say: “But you know that not doing it puts your baby at risk.” And that woman was really like “I do not want to. I do not like needles, I know it takes me out of my concentration.” It was a 5-minute discussion. It went on and on. And then she said: “just do it then”. Afterwards, all her courage was gone. She said: “I cannot handle it, I cannot take the contractions anymore.” She panicked. The needle hurt her and there was nothing they were going to do about it. She said: “I want the needle out.” But they did not do it. It was really… then she was like: “give me an epidural then.” (Doula 3)*

Here, the threat to the baby’s life is seriously unfounded (and not only exaggerated as in certain situations above), since the “needle” is not really needed as a life-saving measure, it is just a precaution, in order to get the mother to comply with the proposed policy. In the quote above, it becomes visible how the constant negotiation of the mother’s consent, and thereby the implicit questioning of her moral and epistemic capacity to give consent, pushes her to give up resistance as an autonomous agent.

## 4. The ethico-epistemic injustice to physical violence pipeline: Epistemic firewalls and conflicting moral understandings

How is it possible that mothers are treated the way they are, presuming that care workers are generally well-meaning individuals who dedicate their lives to the care of others, and why is there not more public outrage about obstetric violence? From the results spring, we believe, two main answers. The first can be understood as a conflicting moral understanding of reproductive justice between the medical establishment and pregnant people, which is a struggle that goes back to the beginning of the obstetric and gynaecological institution. The second is that there is epistemic injustice at play wherein mothers are disregarded as epistemic agents, which makes it seem necessary for doctors to take charge. Epistemic injustice has been understood as an integral part of obstetric violence before [44–47]. But the combination of epistemic injustice with conflicting moral understandings, is particularly dangerous since it makes it possible to disregard people as epistemic agents *and* be either morally ignorant about it or make it morally justifiable to do so. To our knowledge, epistemic injustice in combination with an analysis of, what could be termed, moral injustice, has not been used to understand the widespread and normalized occurrence of obstetric violence before.

The combination of an analysis of epistemic injustice and conflicting moral understandings form the heart of the feminist field of care ethics, ever since Carol Gilligan theorized how girls’ moral reasoning was not understood as moral, and Joan Tronto laid bare the moral boundaries of our epistemic conception of care and politics [48,49]. These two dimensions of injustice have been articulated together most explicitly by Margaret Urban Walker. According to Walker, three processes contribute to the moral and epistemic injustice of making some violence seem normal and “matter of course”: *naturalizing*, *privatizing* and *normalizing* [50–52]. The naturalizing, privatizing, and normalizing of violence through moral and epistemic injustice, constitutes an ‘epistemic firewall’–although it could perhaps better be termed an ethico-epistemic firewall–by ‘sealing off recognizable injuries and credible complaints’ [53]. Hilde Lindemann Nelson described Walker’s concept of the firewall as:

*A barrier that is erected between the privileged and the disenfranchised by various practices that naturalize, normalize, hire, or legitimate coercive behaviors and relations. The firewall makes a state of affairs seem so obvious, so in keeping with the right and good order of things, that the counter story gets dismissed as offensive, tiresome, threatening, or ridiculous. Often, it received no sort of hearing at all. The task, then, is to figure out how to push a counter story through the firewall. [54]*

All three processes of naturalizing, privatizing, and normalizing that are constitutive of an ethico-epistemic firewall when it comes to the practice, justification, and invisibility of obstetric violence, can be recognized in the main themes, playing the dead baby card and troubling consent, that came forth out of our study.

First, *naturalizing* identities is the process of making identities seem “naturally” morally and epistemically disadvantaged, which is something that we saw in the sub-theme “not being asked consent”. Here, it seems natural that the doctors know better than the pregnant people what is the right thing to do; no consent seems to be needed and this is not a moral problem. The epistemic inequality that comes with being pregnant can seem like a natural given within obstetrics. Stella Villarmea has historically traced that being pregnant directly puts one far behind in terms of epistemic injustice, due to the prejudice that the more present the uterus is, the less rational the pregnant subject [55]. And in terms of moral authority, Trudy Dehue has laid bare in her history of pregnancy and abortion in the Netherlands, that moral authority on reproduction was given to gynaecologists and obstetricians in the 1980’s, rather than to women themselves, after it has been in the hands of the church [56]. As such, doctors took up the moral position of priests, she argues, managing access to abortion, whilst leaving pregnant people in a dependent position to make their case convincingly. Our study indicates that the hegemonic moral configuration of what is justice in matters of reproduction, is still in the hands of the obstetric institution, as if pregnant people are *naturally* not only epistemically but also morally less capable of determining what is the right thing to do.

Second, *privatizing* happens when certain treatments or practices are organized in such a way that their scrutiny is prevented:

*Effected by customs, moral understandings, or laws that declare certain interactions outside legitimate or acceptable scrutiny, reaction, or public comment by others, even if those interactions take place in plain sight [57].*

The play of the dead baby card, captured in sub-theme one, contributes significantly to the prevention of scrutiny when it comes to obstetric violence, as it effectively reproduces the moral understanding that the life of the baby is a priority over the life and experiences of the mother. Consequently, violence that happens to the mother is hidden in plain sight, since anything is justified with the supposed rescue of the baby’s life. Dehue laid bare the extensive history of the saving of the life of the child at the cost of the mother’s life in the Netherlands [56]. Until the beginning of the 20^th^ century, there were Catholic guidelines for doctors to make a caesarean section (which was not yet safe at the time) when the foetus was dying during birth in order to baptize it before its death, although this often meant the final blow to the life of the mother. Similarly, until the revision of the abortion law in the late twentieth century, abortions for medical reasons that threatened the life of the mother would not be allowed in the Netherlands, as they are still not in other European countries [56]. Playing the dead baby card can hence be understood as a continuation of a patriarchal moral configuration of “justice” in matters of reproduction, which has for centuries been to prioritize the child above the mother. Although this moral claim can no longer unproblematically be explicitly made, it is, as we saw in the results, still made implicitly. In reaction to the shocking numbers on obstetric violence in the Netherlands, the gynaecologist who co-authored the research that revealed these numbers explained that care workers often intuitively prioritize the interests of the baby above those of the mother, without problematizing this prioritization [58]. We saw the same thing happening in our study in the case of the mother, who merely questioned the way things went during her birth. Here, it was immediately suggested by the doctor that if it would have gone any other way, the baby might have died. That means that even by questioning or exploring other ways the delivery might have gone, or by thinking the birth through step by step, the mother is made to feel as if she puts the baby’s life retrospectively at stake. The mother’s epistemic participation in trying to understand what happened is made impossible, or is rendered illegitimate, based on an implicit moral claim to the baby’s life and a configuration of justice in matters of reproduction that implies her self-sacrifice. The dead baby card is hence not only a ‘threat’ to the baby’s life used to manipulate the mother via the love for her child, but also a moral justification for the dismissal of the mother as an epistemic agent *and* a justification for the dismissal of the moral duty of the doctor to protect the bodily integrity and self-determination of the mother. As a consequence, we arrive in what Elselijn Kingma terms “the paradox of autonomy and consent” where the obstetric patient is at particular risk of being harmed for the promotion of someone else’s well-being, while in similar medical situations (like transplantation or research) an extensive process of informed consent is required [59].

The persistence of this specific configuration of justice in the case of reproduction, is also signalled by De Vries, who argues that it is the bio-ethicist concept of the maternal-foetal conflict wherein the principle of autonomy of the mother is weighed out against the obligation to beneficence to the baby. This continuously reproduces a moral configuration of justice that prioritizes the foetus and makes the mother invisible [60]. The moral prioritization of the child produces a moral ignorance towards the mother. If this was not the case, the prioritization of the child would not look like justice or like “matter of course”, but would be clearly visible as injustice on part of the mother. The moral claim to the life of the child, and the (often exaggerated) threat to it, hence effectively prevents medical and public scrutiny when it comes to the autonomy and self-determination of the mother: The moral need for self-determination of the mother evaporates in plain sight through the shroud waving of the baby. The playing of the dead baby card–either as explicit shroud waving, or implicitly, or as the normalization and justification of obstetric violence–is based on both the moral understanding that it is justified to prioritize the baby’s life over the mother, *and* the epistemic inequality that establishes the authority of the obstetric institution as medical specialists when it comes to saving babies. As a result, the practices that are being called out as obstetric violence, can be kept “private” behind a moral and epistemic firewall, both during medical consultations and in public; for no one else to judge or scrutinize, since the obstetric institution has both the moral and epistemic authority.

Finally, the third process that constitutes the ethico-epistemic firewall is the process of *normalizing* certain patterns of practices. This happens when ‘practices that otherwise would look bad are rendered normal […] for certain contexts or certain people in them’ [53]. When these ‘otherwise bad’ patterns of practice are normalized for ‘certain contexts or certain people in them’, three things happen: first, the presumptions underlying these patterns of behaviour are left unquestioned (e.g. when it is presumed that a person is irrational, the focus shifts from the coercion that they experience to actions that are permitted); second, normalizing then consists in the regulation of the patterns of practice, instead of prohibiting them (under conditions x and y, it is normal to overrule a person’s right to consent); and third, the ones who demand to be heard or have self-control, are discredited [50,51]. All three come back in the results in the theme ‘troubling consent’.

First, since it is normal, and an inheritance of moral and epistemic conceptions of the past, that people in labour are not asked for consent, not asking for consent is left unquestioned–again, in the Netherlands, 42% did not give consent for their episiotomies, 47% not for medication during labour, and 37% did not consent to their ECG monitoring, while 60% of refusals in case of vaginal examinations were overruled [6]. Rather than focusing on this grand scale violation of bodily integrity, the focus in the media, including doctors in the media, was to justify instead of question these patterns, while in any other context, justifying penetrative intimate practices without consent would seem absurd. Second, while juridically pregnant people have the right to bodily self-determination, the violation of self-determination is regulated through moral configurations of when it is fair to overrule someone’s refusal, for instance when the life of the foetus is believed to be at risk. The play of the dead baby card, for instance, functions to morally regulate and justify the negation or ‘troubling’ of consent. Obstetric violence is hence normalized as a practice through a regulatory moral and epistemic troubling of consent: consent is constantly renegotiated, and weakened on the basis of conflicting moral understandings of what is justice in matters of reproduction and epistemic injustice. And third, the people who raise the issue are discredited. This is what happens often to individual parents, but also opinion makers of various major newspapers in the Netherlands condemned the women who participated in research on this subject as ‘wining’ about the people who saved their babies’ lives [14,15]. This last aspect of normalizing bad practices, makes ‘those who rebel against what “everybody” accepts appear as irrational freaks, malcontents, complainers, unstable deviants, or dangerous elements out of control’ [53]. And it makes them, in the case of obstetric violence, into bad or mad mothers who would put their own interests above the life of their child. The effect of the normalization of a bad practice, is that people with claims against this practice, ‘prove’ themselves that they are ‘abnormal’, and even prove ‘their unreliability as judges and informants, and the incredibility of their testimonies’ [50,51,61].

Naturalizing moral and epistemic injustice between pregnant people and those who take care of them, privatizing obstetric violence and preventing public scrutiny through the play of the dead baby card, and normalizing obstetric violence through the troubling of consent, constitute a moral ignorance towards mothers in the form of an ethico-epistemic firewall that bounces back and ridicules maternal quests for knowledge, their questioning of practices, their testimonies of traumatic experiences, their alternative treatment plans, and their attempts at moral and epistemic authority and bodily self-determination. It makes the obstetric institution take moral and epistemic responsibility over the baby, while at the same time negating the mother’s moral and epistemic authority over her child, and her chance to take responsibility. The firewall creates a pipeline from moral and epistemic injustice to physical forms of obstetric violence. Acknowledging this is important for the demystification of obstetric violence: It is not difficult to see how even well-meaning midwives, doctors, and nurses, would appropriate someone’s most intimate body-parts in the unconsented carrying out of their decision, since they believe that they are doing what is best.

That professionals in the obstetric institution do not see the harm they do to pregnant people, is a problem of the normative standpoint from which they think and act in the world. Just as standpoint theory developed an analysis of different epistemic standpoints wherein some are more advantageous than others, Loick [62] discusses how care ethics has developed a similar theory when it comes to moral standpoints. A different social position in the world shapes one’s values, and in this case, one’s moral understanding of, and normative relation to, what is justice. In our case, there are conflicting normative standpoints on what justice is in matters of reproduction. That there can be different moral configurations of justice within different social positions, is a critical feminist insight from the field of care ethics, ever since the ground-breaking work of Carol Gilligan [48]. Gilligan theorized that gender influences the way justice is conceptualized: while children socialized as boys tend to conceptualize justice through abstract principles, girls have a care-based understanding of justice, and determine through an evaluation of context, practice, material dependencies, and affected relationalities, what the ‘good’ is in a specific situation. If we translate this to reproductive justice, it can be, for instance, that while the doctor is unable to morally understand why a mother would take any unnecessary risks with regard to the foetus because of a general moral conception of justice that babies should be born with the least risk possible, the mother can have a plural and relational understanding of justice wherein she is able to want the best for her child *and* the best for herself, because she understands herself and her child as an inseparable sociality of care. These two moral configurations of justice are currently not of equal standing, however, due to the dismissal of mothers as moral epistemic agents. And it is precisely because of the dismissal of mothers as moral epistemic agents due to the ethico-epistemic firewall, that mothers’ normative standpoint should be valued higher when it comes to reproductive justice, since the obstetric institution is stuck in a moral ignorance when it comes to pregnant people that causes an inability to see and understand the harm that obstetric violence causes them. Since mothers’ have a more complete moral understanding of the situation and all actors involved, people with the capacity for pregnancy are hence in a better position to make normative claims when it comes to reproductive justice, just as marginalized people have an epistemic advantage according to standpoint theory. Since the mother is most affected by the situation and has the most epistemic insight on her own life and circumstances, her moral understanding of what is justice in her specific situation is more developed than the doctor’s, the state’s, or society’s configuration of reproductive justice. And this insight has potentially universal reach: It is precisely because the mother is confronted with moral and epistemic injustice and violence, and hence, with the experience of ‘struggle’ [62], that she understands, better than the doctor, that reproductive justice in all situations depends on the beliefs and insights of the mother and should therefore always be primarily morally deliberated by her.

While we cannot change the whole culture within obstetrics at once, and while the elimination of obstetric violence would mean a fundamental reorganization of maternity care and of our moral and epistemic configurations of justice in maternity care, it is possible to resist the moral and epistemic injustice mothers face, and the normalization of obstetric violence on a relational level. This would entail taking mothers radically seriously, also if that makes a health care provider uncomfortable, to uphold their right to autonomy and self-determination, to not manipulate or gaslight mothers into accepting proposed policy, but making sure they are informed in such a way that epistemic inequality between a birth worker and a mother is reduced to a minimum, and to follow pregnant people in their wishes and their concerns by thinking along with them. This is something that every individual health care provider can challenge themselves to do on a daily basis. Truly centring pregnant people will transgress the borders of the current system and protocols, and eventually change it. Rather than normalizing the shroud waving of autonomy and self-determination, the lack of consent and the overruling of refusal, the aim must be to normalize the autonomy of pregnant people to a point that any lack of self-determination and consent is immediately flagged as injustice. On an organizational level this would mean that continuity of care, one-to-one relationships within maternity care, culturally sensitive and personalized care, as well as time, should be priorities in order to facilitate birth workers in their desire to take the people they care for radically seriously.

## Supporting information

S1 File (PDF)

S2 File (PDF)

S3 File (DOCX)
