# Supplementary material for: Shroud waving self-determination: A qualitative analysis of the moral and epistemic dimensions of obstetric violence in the Netherlands
Source: PLoS One. 2024 Apr 22;19(4):e0297968. doi: 10.1371/journal.pone.0297968 (PMC11034656; doi:10.1371/journal.pone.0297968)
Supplement: S1 File — (PDF) [file pone.0297968.s001.pdf]

UNIVERSITEIT VOOR HUMANISTIEK

Verklaring Ethische Toetsingscommissie Universiteit voor Humanistiek

Dossiernummer: 2020-9
Datum: 8 januari 2021

Bij deze verklaar ik dat de Ethische Toetsingscommissie van de Universiteit voor Humanistiek het voorstel voor het door drs. Rodante van der Waal uit te voeren onderzoek getiteld *What is Obstetric Violence? A Critical Study of the Mother-Midwife Relation* heeft getoetst en dat heeft goedgekeurd nadat de onderzoeker het door haar uitgevoerde DPIA op basis van de aanwijzingen van de Privacy Officer van 2 november 2020 naar zijn tevredenheid heeft aangepast en de onderzoeker de respondenten heeft geïnformeerd over manier waarop met de data wordt omgegaan.

Dr. Marie-Christine Opdenakker
Vice-voorzitter Ethische Toetsingscommissie Universiteit voor Humanistiek
