# Supplementary material for: Shroud waving self-determination: A qualitative analysis of the moral and epistemic dimensions of obstetric violence in the Netherlands
Source: PLoS One. 2024 Apr 22;19(4):e0297968. doi: 10.1371/journal.pone.0297968 (PMC11034656; doi:10.1371/journal.pone.0297968)
Supplement: S2 File — (PDF) [file pone.0297968.s002.pdf]

metc.Utrecht

Universiteit voor Humanistiek
t.a.v. Dr. A.A.M. van Nistelrooij
Per e-mail: i.vannistelrooij@uvh.nl

**Ons kenmerk**	WAG/mb/19/021007
**Datum**	19 juni 2019
**Betreft**	METC-protocolnummer 19-411/C<br>Advies niet-WMO onderzoek

Medisch Ethische Toetsingscommissie

Contact:
Afdeling Toetsing Onderzoek

Tel 088 75 563 76
(ochtend ma t/m do)
info@metcutrecht.nl

Geachte heer/mevrouw van Nistelrooij,

De Medisch Ethische Toetsingscommissie (METC), heeft de documenten bij onderzoeksvoorstel nummer 19/411, getiteld **"Controversies surrounding Autonomy in the mother-midwife Relation: a care-Ethical study (ZonMW-projectnummer 854011008)"**, ingediend door dr. C.J.W. Leget, met als verrichter Universiteit voor Humanistiek, ontvangen en afgewogen of toetsing van het voorstel onder de WMO (Wet Medisch Wetenschappelijk Onderzoek met mensen) vereist is.

Het dagelijks bestuur van de METC Utrecht heeft op 18 juni 2019 op basis van de hieronder genoemde documenten geconcludeerd dat het voorstel niet onder de reikwijdte van de WMO valt om één of beide van de volgende redenen:

- Er is géén sprake van medisch-wetenschappelijk onderzoek volgens de definitie van de CCMO. De definitie is te vinden op de pagina van de CCMO website: https://www.ccmo.nl/onderzoekers/wet-en-regelgeving-voor-medisch-wetenschappelijk-onderzoek/uw-onderzoek-wmo-plichtig-of-niet).

en/of

- Personen worden niet aan handelingen onderworpen of hen worden geen gedragsregels opgelegd waarvoor toetsing vereist is.

Dit betekent dat de METC geen wettelijke taak heeft bij de inhoudelijke beoordeling van dit voorstel. De commissie attendeert u er op dat zij alleen heeft afgewogen of

Bezoekadres:
Heidelberglaan 100
3584 CX Utrecht

Postadres:
Huispostnummer D01.343
Kamernummer C01.314
Postbus 85500
3508 GA Utrecht

www.metcutrecht.nl

het voorstel onder de reikwijdte van de WMO valt en daarmee is deze verklaring geen toestemming voor de uitvoer van het voorstel. U bent zelf verantwoordelijk voor de uitvoering van het voorstel volgens de geldende wet- en regelgeving waaronder, maar niet beperkt tot, de AVG (Algemene Verordening Gegevensbescherming), de WGBO en het beleid van de instelling waar het onderzoek wordt uitgevoerd. Het is niet nodig dat u de METC nader informeert over voortgang en afsluiting van het onderzoek

De commissie heeft de volgende documenten in haar afweging meegenomen:

**Onderwerp**	**Datum Ontvangst**
A1. Aanbiedingsbrief dd 17-06-2019	17-6-2019
A1. Formulier (niet-)WMO dd 14-06-2019	17-6-2019

Het is raadzaam wijzigingen en/of addenda waardoor dit voorstel wel onder de reikwijdte van de WMO zou kunnen vallen, aan de METC voor te leggen.

Met vriendelijke groeten,
namens de METC,

Afdeling Toetsing Onderzoek

Aangezien voor deze brief geen wettelijke verplichting tot ondertekening geldt, wordt deze brief zonder handtekening verzonden.

Kopie

- Hoofdonderzoeker: dr. C.J.W. Leget, per e-mail: c.leget@uvh.nl

To whom it may concern,

Referring to our letter of 19 June 2019 (reference number WAG/mb/19/021007) it is hereby confirmed that the Medical Research Involving Human Subjects Act (WMO) does not apply to the above mentioned study and that therefore an official approval of this study by the MREC Utrecht is not required under the WMO.

Yours sincerely,
on behalf of the Medical Research Ethics Committee,

Department of Research Review
