# Supplementary material for: Shroud waving self-determination: A qualitative analysis of the moral and epistemic dimensions of obstetric violence in the Netherlands
Source: PLoS One. 2024 Apr 22;19(4):e0297968. doi: 10.1371/journal.pone.0297968 (PMC11034656; doi:10.1371/journal.pone.0297968)
Supplement: S3 File — (DOCX) [file pone.0297968.s003.docx]

**Statement Ethical Assessment Committee University for Humanistic Studies**

File number: 2020-9

Date: January 8, 2021

I hereby declare that the Ethics Review Committee of the University of Humanistic Studies has approved the proposal for the research to be carried out by drs. Rodante van der Waal entitled “What is Obstetric Violence? A Critical Study of the Mother-Midwife Relationship”

The research was reviewed and approved after the researcher has performed the DPIA based on the instructions of the Privacy Officer of November 2, 2020 to his satisfaction and the researcher the informed respondents about how the data is handled.

Dr. Marie-Christine Opdenakker

Vice-chairman of the Ethics Assessment Committee of the University of Humanistic Studies
